# IGFBP5 promotes diabetic kidney disease progression by enhancing PFKFB3-mediated endothelial glycolysis

**DOI:** 10.1038/s41419-022-04803-y

**Published:** 2022-04-13

**Authors:** Chengcheng Song, Shuqiang Wang, Zhangning Fu, Kun Chi, Xiaodong Geng, Chao Liu, Guangyan Cai, Xiangmei Chen, Di Wu, Quan Hong

**Affiliations:** 1 Medical School of Chinese PLA, Beijing, 100853 China; 2 Department of Nephrology, First Medical Center of Chinese PLA General Hospital, Nephrology Institute of the Chinese People’s Liberation Army, State Key Laboratory of Kidney Diseases, National Clinical Research Center for Kidney Diseases, Beijing Key Laboratory of Kidney Disease Research, Beijing, 100853 China; 3 Department of Nephrology, Peking University Shenzhen Hospital, Shenzhen, 518000 China; 4 Beidaihe Rehabilitation and Recuperation Center, Chinese People’s Liberation Army Joint Logistics Support Force, Qinhuangdao, 066100 China

**Keywords:** Mechanisms of disease, Diabetic nephropathy

## Abstract

Renal inflammation is a critical pathophysiological characteristic of diabetic kidney disease (DKD). The mechanism of the inflammatory response is complicated, and there are few effective treatments for renal inflammation that can be used clinically. Insulin-like growth factor-binding protein 5 (IGFBP5) is an important secretory protein that is related to inflammation and fibrosis in several tissues. Studies have shown that the IGFBP5 level is significantly upregulated in DKD. However, the function of IGFBP5 and its mechanism in DKD remain unclear. Here, we showed that IGFBP5 levels were significantly increased in the kidneys of diabetic mice. Ablation of IGFBP5 alleviated kidney inflammation in DKD mice. Mechanistically, IGFBP5 increased glycolysis, which was characterized by increases in lactic acid and the extracellular acidification rate, by activating the transcription factor early growth response 1 (EGR1) and enhancing the expression of PFKFB3 in endothelial cells. Furthermore, a mutation in PFKFB3 attenuated renal inflammation in DKD mice. Taken together, we provided evidence that IGFBP5 enhanced kidney inflammation through metabolic reprogramming of glomerular endothelial cells. Our results provide new mechanistic insights into the effect of IGFBP5 on kidney and highlight potential therapeutic opportunities for IGFBP5 and the metabolic regulators involved in DKD.

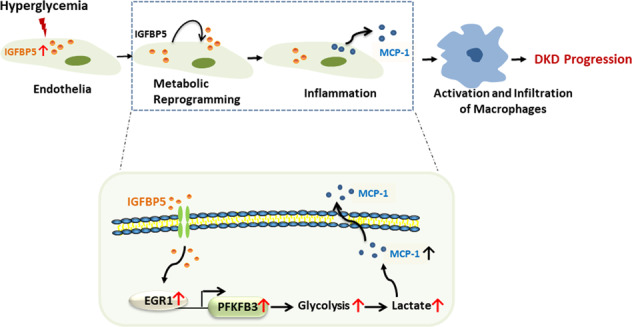

## Introduction

Diabetes is a global epidemic. The International Diabetes Federation reports that 537 million people worldwide suffer from diabetes, and this number is predicted to rise to 643 million by 2030 and 783 million by 2045 [1]. Diabetic kidney disease (DKD), a chronic progressive disease, is currently the major cause of kidney replacement therapy [2]. Metabolic and hemodynamic insults drive the mechanisms involved in the pathophysiology of DKD and its progression toward end-stage renal disease. The multipathogenic factors of DKD and the multiple molecular mechanisms involved may be responsible for the lack of a single treatment with good therapeutic efficacy. Attention should be given to the early pathophysiological changes that occur in DKD, which may be drivers of alterations in kidney structure and function.

There is growing evidence that kidney inflammation plays a vital role in the occurrence and development of DKD [3]. Inflammation and oxidative stress are strongly correlated with the risk of developing DKD [4–6]. Immune cell infiltration (mainly macrophages) of renal tissue is increased in both animal models and patients with DKD [7]. In addition, adhesion molecules and chemokines are upregulated during DKD [8]. These findings reveal the importance of inflammatory mechanisms in promoting diabetes-associated kidney damage.

Glomerular endothelial cells (GECs) have been intensively studied in recent decades [9]. Endothelial activation is a hallmark in diabetes mellitus [10]. GEC dysfunction arises early and plays a key role in the initiation and development of DKD [11, 12]. Diabetes-induced GEC dysfunction is associated with the acquisition of proinflammatory and prothrombotic phenotypes that favor immune cell adhesion and infiltration, the destruction of fenestrated endothelial integrity, and increased endothelial-to-mesenchymal transition [13, 14]. Activation of the endothelial inflammatory phenotype and the synthesis of proinflammatory factors are energy-dependent processes, and metabolic pathways in ECs can reprogram EC phenotypes independently [15]. Studies have shown a relationship between glycolysis and endothelial inflammation. The critical modulator 6-phosphofructose-2-kinase/fructose-2,6-bisphosphatase 3 (PFKFB3) regulates the rate of glycolysis by controlling the level of Fructose-2, 6-bisphosphate (F-2,6-BP), which is considered to be a potent allosteric activator of 6-phosphofructose-1-kinase (PFK1), a key rate-limiting enzyme of glycolysis [16]. The conversion of F-6-P to F-1,6-BP, which is catalyzed by PFK1, is the main rate-limiting step in determining glycolytic flux. PFKFB3-induced glycolysis in ECs mediates the development of angiogenesis and inflammatory diseases such as retinopathy [17].

Insulin-like growth factor-binding protein 5 (IGFBP5) is a secreted protein and an IGF-binding protein that belongs to the IGFBP family (IGFBP1 to IGFBP7), which is a group of proteins that are capable of binding IGF and have two-side effects on IGF I and IGF II. In addition to regulating IGF activity, IGFBPs also have very important functions independent of IGF [18]. Among them, IGFBP5 is the most highly conserved member. Although IGFBP5 mainly regulates the activity of IGF, it has many other biological functions independent of IGF, including the inflammatory response, cell adhesion, cell migration, cell proliferation, and fibrosis [19–22]. Upregulation of IGFBP5 expression was observed in diabetes-related complications, including diabetic neuropathy [23], diabetic heart disease, and diabetic bone disorder [24]. These findings strongly suggest that IGFBP5 has a crucial role in promoting the development of diabetes-related complications. However, the specific mechanism of IGFBP5 in the pathogenesis of DKD remains unclear. Interestingly, the single-cell sequencing database showed that IGFBP5 was highly expressed in GECs from both healthy humans and mice [25, 26], as well as in patients with DKD [27]. Consequently, we hypothesized that IGFBP5 activated the inflammatory response by regulating endothelial metabolism. Therefore, in this work, we detected the role of IGFBP5 in diabetic glomerular injury and provided new targets for the treatment of DKD.

## Materials and methods

### Mouse models

The IGFBP5-knockout (IGFBP5^−/−^) mouse strain on a C57BL/6J background was generated using the CRISPR/Cas9 method (Cyagen Biosciences (Suzhou) Inc., Jiangsu, China). The knockout recognizes a total of 4 exons as target sites, and the transcript code is ENSMUST00000027377. The guide RNA sequences were (gRNA1) ATTCTGACCCGATCCCGCCTGGG and (gRNA2) CCTTTTTGAAGCAAGTTGAACGG. Successful mutation of the IGFBP5 gene was identified by PCR genotyping using tail DNA. PFKFB3-Mut knock-in C57BL/6J mice (PFKFB3^wt/mut^) were a gift from Professor Li (Xiamen University, China) [28]. db/db (BKS-Lepr^em2Cd479^/Gpt) mice and age-matched non-diabetic db/m male mice were purchased from GemPharmatech Co., Ltd. (Jiangsu, China). The experimental animals were randomly assigned to each group. All mice were kept in specific pathogen-free facilities and housed with 12 h cycles of light/darkness and a standard chow diet at 25 °C. Animal experiments were approved by the Ethics Committee of PLA General Hospital.

#### STZ-induced diabetic murine model

At 8 weeks of age, the male mice were administered streptozotocin (STZ, Sigma-Aldrich, St. Louis, MO, USA) by intraperitoneal injection after a 6-h fast for 5 consecutive days (50 mg/kg/day). One week after the last injection, the blood glucose levels of the mice were measured. Diabetes was confirmed by a fasting blood glucose level >300 mg/dl. Age- and sex-matched mice were injected with citrate vehicle served as controls. All mice were euthanized at 12 or 16 weeks after STZ or vehicle injection. Body weight and blood glucose levels were monitored biweekly. Random spot urine was collected to measure urinary protein and creatinine. Kidney tissues were fixed in 4% paraformaldehyde for pathological analysis.

#### Overexpression of IGFBP5 in diabetic mice

To overexpress IGFBP5 in the kidneys of mice, recombinant lentivirus expressing a murine IGFBP5 coding sequence, which was synthesized by Fenghui Biotechnology (Hunan, China), was injected through the tail vein. Mice in the control group were injected with scramble lentivirus (vehicle). The mice were treated with IGFBP5 overexpression (IGFBP5-OE) vectors once each week for 2 weeks and sacrificed, and samples were collected.

#### Pharmacological inhibition of PFKFB3 in diabetic mice

db/db mice were intraperitoneally injected with 3-(3-pyridinyl)-1(4-pyridinyl)-2-propen-1-one (3PO) (50 mg/kg, 4×/wk) or dimethyl sulfoxide (DMSO) (vehicle) for 4 consecutive weeks [29]. 3PO and DMSO were purchased from Sigma-Aldrich (Louis, MO, USA). Glucose, body weight, urinary microalbumin, and urinary creatinine were measured weekly. Kidney tissues were fixed in 4% paraformaldehyde for pathological analysis.

### Cell culture

Human umbilical vein endothelial cells (HUVECs) and human monocytic THP-1 cells were purchased from the American Type Culture Collection (ATCC). HUVECs were cultured in endothelial cell medium (ECM, ScienCell, USA). THP-1 cells were cultured in Dulbecco’s modified Eagle’s medium (DMEM, Gibco, USA) supplemented with fetal bovine serum (10%, FBS, Gibco, USA). Cells were maintained in a humidified incubator with 5% CO_2_ at 37 °C.

### RNA-sequencing (RNA-seq) analysis

IGFBP5-OE HUVECs and normal HUVECs were seeded on six-well culture plates and grown to ~70% confluence in complete medium for 12 h, after which the medium was changed to glucose- and serum-free medium, and the cells were incubated for 6 h. The normal cells were then exposed to normal glucose (NG, 5.5 mmol/l) and high glucose (HG, 30 mmol/l) in complete medium for 48 h. IGFBP5-OE cells were cultivated in NG conditions for 48 h. Total RNA was collected using TRIzol reagent (Invitrogen, Grand Island, NY) for subsequent RNA-sequencing analysis.

### siRNA-mediated knockdown

siRNAs targeting IGFBP5 or PFKFB3 (siIGFBP5 or siPFKFB3, respectively) and a nontargeting negative control (siCTRL) were synthesized by GenePharma (Shanghai, China). siRNAs were transfected into cells using jetPRIME™ (Polyplus-transfection, Strasbourg, France) according to the manufacturer’s instructions. After 24 h, the cells in the different treatment groups were harvested for subsequent assays. The siRNA sequences used are shown in Table S1.

### Quantitative real-time PCR (qRT–PCR)

Total RNA was isolated from cells or kidney tissue using TRIzol reagent (Invitrogen). One microgram of RNA was used to synthesize cDNA with the SuperScript III First-Strand Synthesis System (Applied Biosystems, USA). Real-time PCR was performed (Bio-Rad, Hercules, CA) with FastStart Universal SYBR Green Master Mix (Roche, Mannheim, Germany). The relative mRNA level was determined via the 2^−ΔΔCt^ method and normalized to 18S rRNA. The gene primers were synthesized by BGI (Shenzhen, China) and are listed in Table S2.

### Chromatin immunoprecipitation (ChIP)

The ChIP-PCR procedure was similar to previously described procedures [30]. ChIP DNA from anti-EGR1 (ab55160, Abcam)-treated cells was used to examine the association between EGR1 and PFKFB3. DNA from anti-IgG antibody-treated cells served as the control. Purified DNA was used to analyze the PFKFB3 proximal promoter region by real-time PCR. The PFKFB3 primers were as follows: forward: 5′-TGTGAAAACCAGATGCCAGC-3′; and reverse: 5′-GGACTTGAACTGAGCCTTGC-3′. The relative amplification of the promoter sequence of the gene was calculated using the 2^−ΔΔCT^ method.

### Dual-luciferase reporter assay

The wild-type human PFKFB3 (GenBank 5209) promoter, including the potential binding site (−694 to −682 nt), was obtained by PCR amplification, subcloned into the pGL3-Basic vector (Promega, Madison, USA), and named pGL3-WT-PFK. Additionally, the binding site -TTCCCGCCCCCTCC- within the promoter of PFKFB3 was mutated to -GGACCATCAACGCA- and named pGL3-MT-PFK. Plasmid (pReceiver-M94) containing full-length human EGR1 cDNA was obtained from GeneCopoeia. The EGR1 overexpression plasmid and pGL3WT-PFK or pGL3-MT-PFK were transfected into HUVECs with Lipofectamine 2000 (Invitrogen, Carlsbad, CA, USA). The samples were then cotransfected with a pRL-TK (Promega) plasmid expressing Renilla luciferase. Firefly and Renilla luciferase activity levels were examined using a dual-luciferase reporter assay system.

### Western blotting

Tissues were lysed in RIPA buffer (Beyotime, Shanghai, China) containing 1 mmol/l PMSF, and the protein concentrations were determined with a BCA protein assay kit (Thermo, Rockford, IL, USA). Thirty micrograms of protein was separated by 10% SDS–PAGE (Bio-Rad Laboratories, Hercules, CA, USA) and then transferred to NC membranes. After being incubated with blocking buffer (5% nonfat dry milk in TBST), the membranes were incubated overnight at 4 °C with the following primary antibodies: PFKFB3 (13763-1-AP, Proteintech), IGFBP5 (55205-1-AP, Proteintech), EGR1 (ab55160, Abcam), β-actin (ab8226, Abcam), and tubulin (66240-1, Proteintech).

### Measurement of the extracellular acidification rate (ECAR)

A Seahorse XFe24 Extracellular Flux Analyzer (Agilent Technologies, Inc., Santa Clara, CA) was used to measure the ECAR of the medium. Briefly, HUVECs and IGFBP5-OE cells were seeded in an XF 24-well cell culture microplate at a density of 3 × 10^4^ cells/well and washed in glucose-free XF base medium. The ECAR was measured after serial injections of 100 mmol/l D-glucose, 10 mmol/l oligomycin, and 500 mmol/l 2-deoxyglucose.

### Measurements of glycolytic metabolites

Intracellular F-2,6-BP levels were measured in HUVECs and IGFBP5-OE cells using Van Schaftingen’s method [31]. Cells were incubated with NG or HG medium for 48 h. Then, the cells were homogenized in extraction buffer containing 0.1 mol/l NaOH and 0.1% Triton X-100. The resulting mixture was heated for 5 minutes at 80 °C. After being cooled, the cells were centrifuged, and the supernatant was neutralized with acetic acid. The mixture was centrifuged, and the F-2,6-BP level in the supernatant was measured using a coupled enzyme reaction. The levels of lactate or pyruvate were measured with lactate or pyruvate assay kits (Nanjing Jiancheng, Jiangsu, China), respectively, according to the manufacturer’s instructions.

### Monocyte migration assay

The migratory ability of THP-1 cells was determined using Transwell migration chambers (Corning, 8.0 μm, MA, USA). The number of THP-1 cells in the upper chamber was 2 × 10^5^/well and the number of HUVECs in the lower chamber was 15 × 10^5^/well. After 36 h of coculture, the upper chambers were removed and fixed with 4% paraformaldehyde. Cells on the upper side of the filters were removed with cotton-tipped swabs. Cells that migrated to the lower surface were stained with 0.1% crystal violet (Solarbio, Beijing, China) for 15 min. The number of migrating cells was recorded with ImageJ software. The numbers of cells were counted in five random fields and averaged.

### Measurements of urinary albumin and urine creatinine

The urine albumin concentration was measured using a mouse albumin ELISA kit (Bethyl Laboratories, Montgomery, TX, USA). Urine creatinine levels in the same samples were measured using a creatinine colorimetric assay kit (Cayman, MI, USA) according to the manufacturer’s instructions.

### Kidney histologic and morphometric analyses

Kidney samples were fixed in 10% formalin, embedded in paraffin, and sectioned to 2 μm thickness. The sections were stained with periodic acid–Schiff (PAS) to analyze the glomerular area and mesangial matrix expansion as previously described [32]. Histopathological damage was evaluated and recorded using a light microscope (400× magnification; Olympus, Tokyo, Japan). The mean glomerular tuft volume was determined from the mean glomerular cross-sectional area by light microscopy.

### Immunofluorescent staining

CD68 (ab125212, Abcam, Cambridge, UK) and CD206 (ab64693, Abcam) were examined in paraffin-embedded sections of mouse kidney tissues. Heat-mediated antigen retrieval was performed in citrate buffer (pH 6, epitope retrieval solution) for 10 min. The tissue sections were blocked with 10% goat serum. The tissue sections were then incubated with the previously listed primary antibodies overnight at 4 °C. Cy3-conjugated goat anti-rabbit IgG was used as a secondary antibody at a 1:300 dilution and incubated for 1 h at room temperature. The sections were counterstained with DAPI. The slides were imaged by confocal fluorescence microscopy (Olympus, Tokyo, Japan). Each experiment was repeated three times.

### Statistical analysis

The data are expressed as the mean ± standard deviation, and all experiments were performed at least three independent times. All statistical analyses were performed using GraphPad Prism 8.21 software (La Jolla, CA, USA). The data were analyzed using Student’s *t* test for comparisons between two groups. Statistical differences between three or more groups were evaluated using one-way ANOVA. *P* < 0.05 was considered to indicate a significant difference.

## Results

### IGFBP5 is increased in ECs by high glucose exposure and induces endothelial inflammation

**Fig. 1:**
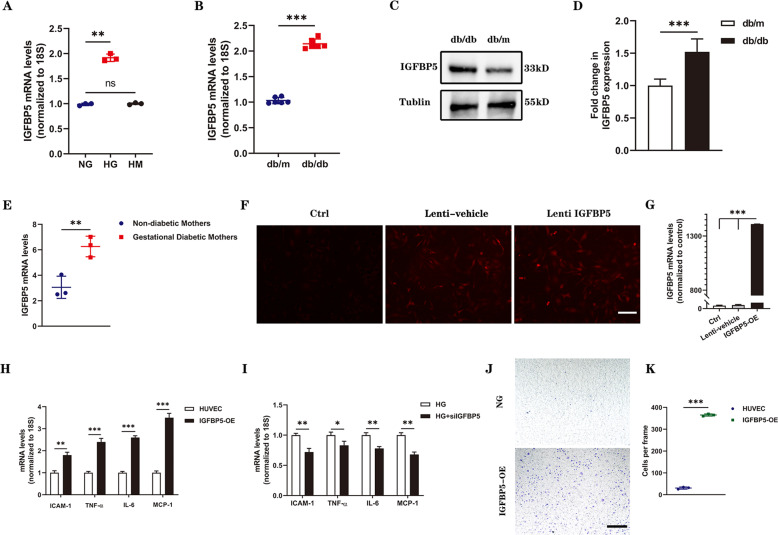
IGFBP5 expression was increased in DKD mice and promoted endothelial inflammation. **A** RT–PCR analysis of IGFBP5 mRNA levels in NG- or HG-challenged HUVECs. *n* = 3. **B** IGFBP5 mRNA was determined by RT–PCR in the kidney tissues of db/db and db/m mice. *n* = 6. **C**, **D** Western blot analysis and densitometric quantification of IGFBP5 protein levels in the kidney lysates of db/m and db/db mice. *n* = 6. **E** RNA-sequencing analysis of IGFBP5 expression in HUVECs isolated from gestational diabetic mothers and non-diabetic mothers. *n* = 3. **F** A stable IGFBP5 overexpression (IGFBP5-OE) HUVEC line was established using lentiviral infection and an IGFBP5 overexpression sequence. Autofluorescence (red) showed successful transfection. Scale bar, 100 μm. **G** Expression of IGFBP5 mRNA in IGFBP5-OE HUVECs compared with the control. *n* = 3. **H** RT–PCR analysis of the mRNA levels of ICAM-1, TNF-α, IL-6, and MCP-1 in IGFBP5-OE and control cells under NG conditions. *n* = 3. **I** RT–PCR analysis of the mRNA levels of ICAM-1, TNF-α, IL-6, and MCP-1 in siIGFBP5-treated HUVECs under HG conditions. *n* = 3. **J** Migration of THP-1 cells cocultured in Transwell systems with IGFBP5-OE or control HUVECs under NG conditions. Scale bar, 100 μm. **K** Statistical analysis was performed by counting the number of migrated cells in different groups. *n* = 3. The data are shown as the mean ± SD in all statistical graphs. *P < 0.05, **P < 0.01, ***P < 0.001; ns, no significance.

HUVECs were cultured with high glucose (HG, 30 mmol/l), normal glucose (NG, 5.5 mmol/l), or high mannitol (HM, 5.5 mmol/l glucose, and 24.5 mmol/l mannitol) medium, and the levels of IGFBP5 were assessed. The expression of IGFBP5 increased under HG conditions, as confirmed by qRT–PCR analysis (Fig. 1A). Next, we measured the expression of IGFBP5 in the kidney tissue of db/db mice by qRT–PCR and found that it was significantly increased compared with that in control mice (db/m) (Fig. 1B). Furthermore, IGFBP5 protein expression was examined in mouse kidneys and was markedly enhanced in db/db mice (Fig. 1C, D). Based on previously published transcriptional sequencing data, the mRNA level of IGFBP5 was increased in HUVECs in gestational diabetes compared to control HUVECs obtained from non-diabetic mothers [33] (Fig. 1E).

Hyperglycemia-induced cell injury induces cells to produce proinflammatory factors, such as adhesion molecules and chemokines, leading to the recruitment of inflammatory cells to kidney, including macrophages [34]. It has been reported that IGFBP5 can induce monocyte migration and exerts profibrotic activity [19, 35]. Therefore, we hypothesized that IGFBP5 could promote endothelial inflammation. We overexpressed IGFBP5 (IGFBP5-OE) in HUVECs by lentivirus (Fig. 1F, G) and then examined the expression levels of inflammatory cytokines. The mRNA levels of proinflammatory cytokines, including IL-6 (interleukin-6) and TNF-α (tumor necrosis factor-α), the chemokine MCP-1 (monocyte chemoattractant protein-1), and the adhesion molecule ICAM-1 (intercellular adhesion molecule-1) in HUVECs (control) and IGFBP5-OE cells under NG conditions were evaluated. The levels of these proinflammatory molecules, chemokines, and adhesion molecules in IGFBP5-OE cells were significantly higher than those in control cells (Fig. 1H). Furthermore, we also measured the mRNA levels of these factors in HUVECs transfected with siCTRL or siIGFBP5 under HG conditions, and these factors were markedly decreased in siIGFBP5-treated cells compared with siCTRL-treated cells (Fig. 1I). To determine whether IGFBP5-induced EC activation increased monocyte recruitment, we designed a Transwell system to study the migration of THP-1 cells in the presence of HUVECs or IGFBP5-OE cells in NG conditions. When THP-1 cells were cocultured with IGFBP5-OE cells, THP-1 cells exhibited enhanced migratory potential (Fig. 1J, K).

These results indicate that IGFBP5 promotes the expression of chemokines in ECs and the migration of macrophages to the glomerulus, supporting our hypothesis that IGFBP5 regulates inflammatory programming in the injured endothelium. Overall, these data show that the increased IGFBP5 expression is related to DKD progression and that IGFBP5 induces a proinflammatory response in ECs.

### Genetic deletion of IGFBP5 attenuates diabetes-induced glomerulopathy and renal inflammation

**Fig. 2:**
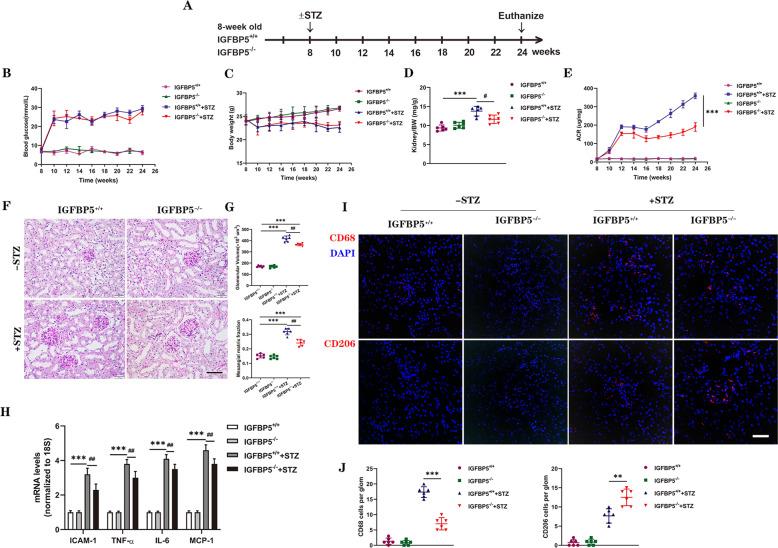
IGFBP5 ablation protects against diabetic glomerulopathy. **A** Schematic showing the experimental design. STZ or citrate buffer vehicle was injected into 8-week-old IGFBP5^+/+^ and IGFBP5^−/−^ mice. The mice were euthanized at 16 weeks postinjection for analysis. *n* = 6 mice. **B**, **C** Biweekly blood glucose and body weight measurements of control and diabetic mice. **D** Kidney to-body weight (BW) ratio at 16 weeks after DM induction. **E** Urinary albumin-to-creatinine ratio (ACR) over time; *n* = 6 mice. **F** Representative images of periodic acid-Schiff (PAS)-stained kidneys. Scale bar, 50 μm. **G** Quantification of glomerular volume and mesangial matrix fraction per mouse; *n* = 6 mice. **H** RT–PCR analysis of the mRNA levels of ICAM-1, TNF-α, IL-6, and MCP-1 in control and diabetic mice. *n* = 6 mice. **I** Representative images of CD68 and CD206 immunofluorescence. Scale bar, 50 μm. **J** Quantification of CD68- and CD206-positive cells in diabetic and non-diabetic IGFBP5^+/+^ or IGFBP5^−/−^ mice. The data are shown as the mean ± SD. **P < 0.01, ***P < 0.001^; #^*P* < 0.05, ^##^*P* < 0.01.

To determine the role of IGFBP5 in DKD, we examined the effects of IGFBP5 loss using global IGFBP5-null (IGFBP5^−/−^) mice. IGFBP5^−/−^ mice were normal as reported previously [36]. As shown in Fig. 2A, diabetes was induced by STZ injections in IGFBP5^−/−^ mice and IGFBP5^+/+^ controls (+STZ). Citrate buffer vehicle-injected mice served as non-diabetic controls (-STZ), and all mice were euthanized at 16 weeks after DM induction. The extent of hyperglycemia and body weight loss were similar between diabetic IGFBP5^+/+^ and IGFBP5^−/−^ mice and non-diabetic mice (Fig. 2B, C). However, STZ-induced kidney hypertrophy was observed in diabetic IGFBP5^+/+^ mice, as determined by the kidney-to-body weight ratio (Fig. 2D). These data suggest that IGFBP5 knockout can improve physiological parameters independent of blood glucose in diabetic mice. The extent of albuminuria, as assessed by the albumin-to-creatinine ratio (ACR) of random urine samples over 16 weeks after STZ, was markedly attenuated in diabetic IGFBP5^−/−^ mice compared with diabetic IGFBP5^+/+^ mice (Fig. 2E). Histologic analysis showed that the development of glomerular hypertrophy and mesangial expansion was significantly blunted in diabetic IGFBP5^−/−^ mice compared with diabetic IGFBP5^+/+^ mice (Fig. 2F, G). IGFBP5 deficiency ameliorated STZ-induced increases in the kidney/body weight ratio and urinary albumin in mice, indicating functional protection of the kidney by IGFBP5 knockout.

The in vitro experiments showed that IGFBP5 regulated the expression of proinflammatory molecules. Thus, we examined markers of kidney inflammation in IGFBP5^−/−^ mice. As expected, the expression of proinflammatory factors increased significantly in diabetic mice (Fig. 2H). However, the inflammatory response in diabetic IGFBP5^−/−^ mice was weakened compared to that in diabetic IGFBP5^+/+^ mice (Fig. 2H). Consistent with this finding, glomerular CD68^+^ macrophages were increased in diabetic IGFBP5^+/+^ mice but were decreased in diabetic IGFBP5^−/−^ mice at 16 weeks after STZ (Fig. 2I, J). However, CD206^+^ macrophages in the glomerulus showed the opposite trend (Fig. 2I, J). Collectively, these data indicate that IGFBP5 is crucial to the development and progression of DKD and validate the promoting effects of IGFBP5 on the transformation of ECs from a normal state to an inflammatory phenotype and the renoprotective effects of IGFBP5 inhibition on diabetic mice.

### IGFBP5 regulates the expression of PFKFB3 via EGR1

**Fig. 3:**
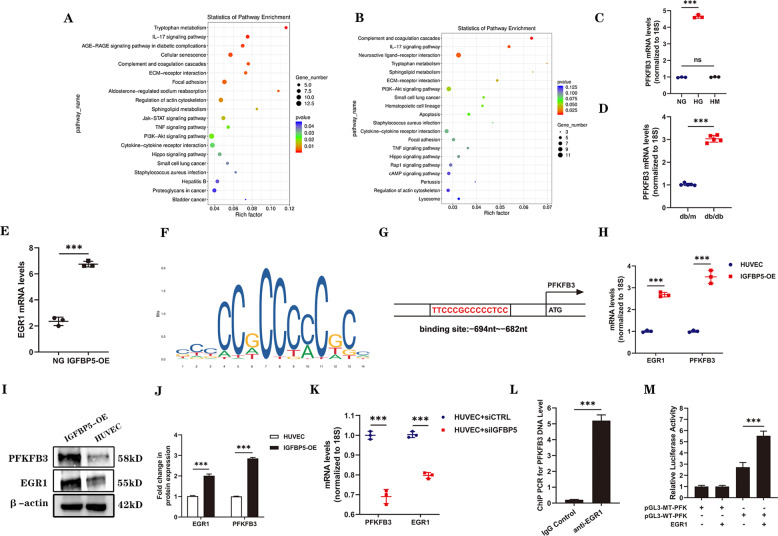
IGFBP5 regulates the expression of PFKFB3 by regulating the binding EGR1 of to PFKFB3. RNA-seq analysis was performed on IGFBP5-OE- and HG-stimulated HUVECs. **A** KEGG pathway analysis of upregulated signaling pathways in IGFBP5-OE cells. **B** KEGG pathway analysis of upregulated signaling pathways in HG-stimulated HUVECs. **C**, **D** RT–PCR analysis of the mRNA levels of PFKFB3 in HG-treated HUVECs and the kidneys of db/db or db/m mice. *n* = 3. **E** RNA-sequencing data of EGR1 expression in IGFBP5-OE cells. *n* = 3. **F** The consensus EGR1-binding motif identified by WebLogo in the EGR1 ChIP-Seq data. **G** The target region of the PFKFB3 promoter to which EGR1 binds. **H** RT–PCR analysis of EGR1 and PFKFB3 mRNA levels in IGFBP5-OE cells. *n* = 3. **I**, **J** Western blot analysis and densitometric quantification of EGR1 and PFKFB3 protein levels in IGFBP5-OE cells under NG conditions. *n* = 3. **K** RT–PCR analysis of EGR1 and PFKFB3 mRNA levels in HUVECs transfected with siIGFBP5 under HG conditions. **L** ChIP-PCR results for PFKFB3. *n* = 3. **M** Dual-luciferase reporter assay results. *n* = 3. The data are shown as the mean ± SD. ***P < 0.001; ns, no significance.

To further confirm the mechanism of IGFBP5 in DKD, RNA sequencing (RNA-seq) was performed to investigate changes in the gene expression profiles of IGFBP5-OE cells or HUVECs stimulated with HG. As shown in Fig. 3A, B, the RNA-seq results showed that the majority of genes involved in inflammatory and metabolic pathways were upregulated in the IGFBP5-OE group and HG group compared with the NG group. This result indicated that cell metabolic reprogramming was involved in the effect of IGFBP5 on DKD. In recent years, a growing number of studies have reported the key role of PFKFB3 in metabolic reprogramming [37–39], and the expression of PFKFB3 was upregulated in ECs exposed to HG conditions (Fig. 3C, D). Factor early growth response 1 (EGR1), which was increased in IGFBP5-OE HUVECs based on RNA-seq data (Fig. 3E), contained 3 C2H2-type zinc fingers (bioinfo.life.hust.edu.cn) (Fig. 3F) and was predicted to have potential binding capacity with PFKFB3 (https://maayanlab.cloud/). Transcription factor prediction online tools (jaspar.genereg.net) indicated that the promoter sequence of PFKFB3 contained a C2H2 zinc finger factor motif at −694 ~ −682 nt (5′-TTCCCGCCCCCTCC-3′) (Fig. 3G). These results suggested that PFKFB3 might be the target of EGR1. EGR1, as an important transcription factor, has been shown to participate in DKD by enhancing ECM production by interacting with TGF-β and contributing to proinflammatory responses in diabetic atherosclerosis [40, 41].

Next, we measured the expression of EGR1 and PFKFB3 in IGFBP5-OE cells and HUVECs under NG conditions. IGFBP5-OE increased the mRNA and protein levels of EGR1 and PFKFB3 (Fig. 3H–J). HUVECs that were pretreated with siIGFBP5 or siCTRL were incubated in HG conditions for 48 h. IGFBP5 knockdown significantly reduced the mRNA expression of EGR1 and PFKFB3 (Fig. 3K). ChIP-PCR showed typically higher expression of PFKFB3 in the anti-EGR1 antibody group than in the background control group, confirming that EGR1 bound to the PFKFB3 promoter (Fig. 3L). Furthermore, we performed a dual-luciferase reporter assay to confirm the targeting relationship between PFKFB3 and EGR1. The wild-type PFKFB3 promoter group of HUVECs showed higher luciferase activity than the pGL3 vector and mutant groups, and promoter activity was increased by EGR1 overexpression (Fig. 3M). The increase in luciferase activity was reversed by transfection with a plasmid expressing the mutant promoter region (Fig. 3M). Taken together, these data suggest that PFKFB3 is a direct transcriptional target of EGR1.

### IGFBP5 regulates the glycolysis required for EC proinflammatory phenotypes via PFKFB3

**Fig. 4:**
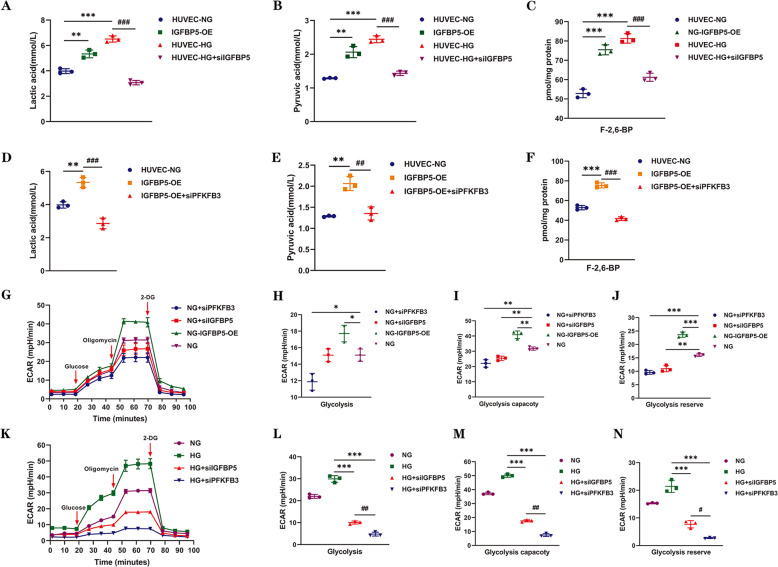
IGFBP5 regulates glycolysis, which is required for endothelial cells, via PFKFB3. Four groups were created. IGFBP5-OE cells and control HUVECs were cultured under NG conditions. HUVECs were cultured in HG conditions and transfected with or without siIGFBP5. The levels of lactic acid (**A**), pyruvate (**B**), and F-2,6-BP (**C**) were measured in each group. *n* = 3. The levels of lactic acid (**D**), pyruvate (**E**), and F-2,6-BP (**F**) were measured in control and IGFBP5-OE cells that were transfected with or without siPFKFB3 under NG conditions. *n* = 3. **G** Glycolytic flux analysis of NG, NG + siPFKFB3, NG + siIGFBP5, and NG + IGFBP5-OE cells by a Seahorse Flux Analyzer. The extracellular acidification rate (ECAR) was recorded after the injection of glucose, oligomycin, and 2-deoxyglucose (2-DG). **H**–**J** Statistical analysis of glycolysis, the glycolytic capacity, and glycolytic reserves in the ECAR. **K** Glycolytic flux analysis of NG, HG, HG + siPFKFB3, and HG + siIGFBP5 HUVECs by a Seahorse Flux Analyzer. The ECAR was recorded after the injection of glucose, oligomycin, and 2-DG. **L**–**N** Statistical analyses of glycolysis, the glycolytic capacity, and glycolytic reserves in the ECAR. The data are shown as the mean ± SD in all statistical graphs. *P < 0.05, **P < 0.01, ***P < 0.001; ^#^*P* < 0.05, ^##^*P* < 0.01, ^###^*P* < 0.001.

Phenotypic reprogramming and inflammation are energy-dependent processes that require underlying metabolic changes to support transcriptional and posttranscriptional changes in gene expression [42]. Therefore, we evaluated metabolic changes in ECs. Lactic acid, a typical metabolite of glycolysis, was examined in HUVECs and IGFBP5-OE cells under NG conditions. The data showed that IGFBP5-OE facilitated the synthesis of lactic acid (Fig. 4A). As expected, the level of lactate was significantly decreased when HUVECs were treated with siIGFBP5 (Fig. 4A). Pyruvate also showed similar metabolic changes (Fig. 4B). As one of the key enzymes in glycolysis, PFKFB3 mainly promotes the synthesis of F-2,6-BP, which promotes the activity of the glycolysis enzyme PFK-1. Thus, we then measured the level of F-2,6-BP in each group and found that IGFBP5 overexpression increased the synthesis of F-2,6-BP, while knockdown had the opposite effect (Fig. 4C). These results suggest that IGFBP5 is involved in the regulation of glycolysis in ECs.

To clarify the role of PFKFB3 in IGFBP5-mediated promotion of glycolysis, we transfected IGFBP5-OE cells with siPFKFB3 and found that the glycolysis-promoting effect of IGFBP5 was strongly inhibited by PFKFB3 knockdown (Fig. 4D–F). This finding further confirmed that IGFBP5 promoted glycolysis by strengthening the expression of PFKFB3. Furthermore, we measured the extracellular acidification rate (ECAR) in ECs with or without IGFBP5/PFKFB3 knockdown or IGFBP5-OE under NG (Fig. 4G–J) or HG (Fig. 4K–N) conditions using the Seahorse XF Glycolytic Pressure Assay. Strikingly, IGFBP5 or PFKFB3 silencing decreased the rates of extracellular acidification in HUVECs, whereas IGFBP5-OE increased the rates (Fig. 4G, K). It is worth noting that siPFKFB3 more robustly inhibited the ECAR than siIGFBP5 under HG conditions (Fig. 4L–N). These data reveal that IGFBP5 promotes glycolysis in HUVECs by upregulating the expression of PFKFB3. In support of this finding, silencing IGFBP5 or PFKFB3 markedly decreased the levels of F-2,6-BP, lactic acid, and pyruvate.

**Fig. 5:**
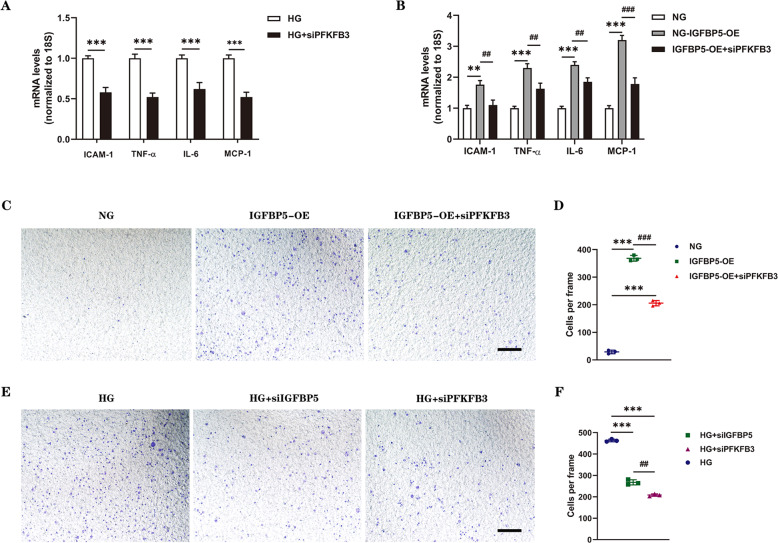
PFKFB3 deficiency inhibits endothelial inflammation and macrophage migration. **A** RT–PCR analysis of the mRNA levels of ICAM-1, TNF-α, IL-6, and MCP-1 in HUVECs transfected with or without siPFKFB3 under HG conditions. *n* = 3. **B** RT–PCR analysis of the mRNA levels of ICAM-1, TNF-α, IL-6, and MCP-1 in control HUVECs and IGFBP5-OE cells with or without siPFKFB3 treatment under NG conditions. *n* = 3. **C** Migration of THP-1 cells that were cocultured in Transwell systems with control or IGFBP5-OE cells (with or without siPFKFB3 involved) in NG conditions for 36 h. Scale bar, 100 μm. **D** Statistical analysis was performed by counting the number of migrated cells in the different groups. *n* = 3. **E** Migration of THP-1 cells that were cocultured in Transwell systems with HUVECs (transfected with siCTRL, siIGFBP5, or siPFKFB3) under HG conditions for 36 h. Scale bar, 100 μm. **F** Statistical analysis was performed by counting the number of migrated cells in the different groups. *n* = 3. The data are shown as the mean ± SD in all statistical graphs. **P < 0.01, ***P < 0.001; ^##^*P* < 0.01, ^###^*P* < 0.001.

To examine the role of PFKFB3 in the regulation of endothelial inflammation in vitro, the mRNA levels of IL-6, TNF-α, ICAM-1, and MCP-1 were evaluated in ECs. The results showed that the mRNA levels of these key molecules were decreased in the siPFKFB3 group compared with the control group (Fig. 5A). Similar results were also observed in IGFBP5-OE cells (Fig. 5B). Thus, inhibiting PFKFB3 in ECs reversed the inflammatory signature. Correspondingly, we investigated the role of PFKFB3 in regulating monocyte migration in vitro, and a Transwell system was used. IGFBP5-OE significantly enhanced the migration of THP-1 cells, and this effect was blocked by endothelial PFKFB3 knockdown (Fig. 5C, D). The results showed that HG significantly promoted monocyte migration, while migration was dramatically suppressed by siPFKFB3 or siIGFBP5 (Fig. 5E, F). These results indicated that endothelial PFKFB3 regulated the production of proinflammatory molecules and promoted endothelial inflammatory reprogramming.

The correlation between endothelial inflammation and metabolic reprogramming was confirmed by the consistency in the expression of proinflammatory factors and chemokines and the trends in glycolytic products and the ECAR. Collectively, these data suggest that IGFBP5 promotes endothelial inflammation by activating PFKFB3-mediated glycolysis.

### A mutation in PFKFB3 reduces endothelial inflammation and macrophage infiltration in the kidney tissues of diabetic mice

**Fig. 6:**
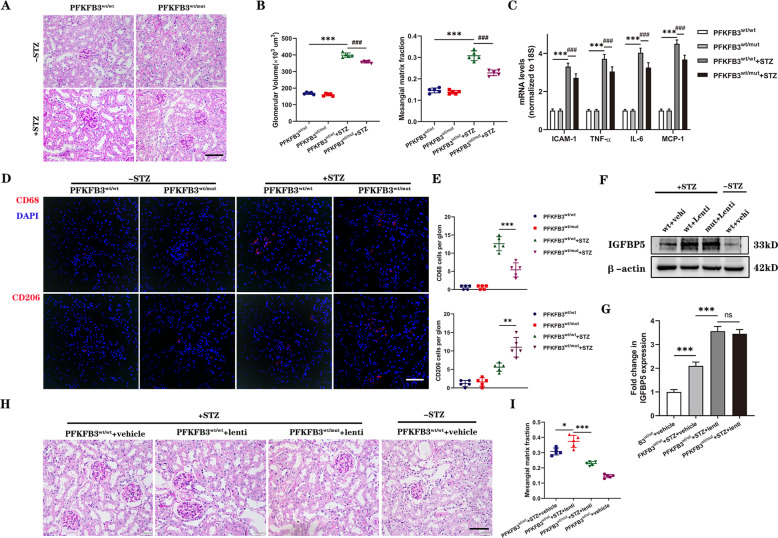
A mutation in PFKFB3 blocks the expression of proinflammatory molecules and the migration of macrophages in the kidneys of diabetic mice. **A** Representative images of PAS-stained kidneys. Scale bar, 50 μm. **B** Quantification of glomerular volume and mesangial matrix fraction per mouse. *n* = 5–6 mice. **C** RT–PCR analysis of the mRNA levels of ICAM-1, TNF-α, IL-6, and MCP-1 in control and diabetic mice. *n* = 5–6 mice. **D** Representative images of CD68 and CD206 immunofluorescence. Scale bar, 50 μm. **E** Quantification of CD68- and CD206-positive cells in diabetic and non-diabetic PFKFB3^wt/mut^ or PFKFB3^wt/wt^ mice. **F**, **G** Western blot analysis and densitometric quantification of IGFBP5 protein levels in the kidney lysates of PFKFB3^wt/wt^ + vehicle, diabetic PFKFB3^wt/wt^ + vehicle, diabetic PFKFB3^wt/wt^ + lentivirus, and diabetic PFKFB3^wt/mut^ + lentivirus mice. *n* = 5 mice. **H** Representative images of PAS-stained kidneys. Scale bar, 50 μm. **I** Quantification of the mesangial matrix fraction per mouse. *n* = 5 mice. The data are shown as the mean ± SD. *P < 0.05, **P < 0.01, ***P < 0.001; ^**###**^*P* < 0.001; ns, no significance.

To confirm whether a mutation in PFKFB3 could suppress renal inflammation in DKD, we used PFKFB3-Mut knock-in mice. The induction of diabetes in PFKFB3-Mut mice was similar to that in IGFBP5^−/−^ mice. Histologic analysis showed that the development of glomerular hypertrophy and mesangial expansion was significantly blunted in diabetic PFKFB3^wt/mut^ mice compared with diabetic PFKFB3^wt/wt^ mice (Fig. 6A, B). We next examined the expression of inflammatory cytokines in PFKFB3^wt/mut^ mice. Consistent with data from IGFBP5^−/−^ mice (Fig. 2H), the levels of IL-6, TNF-α, ICAM-1, and MCP-1 were significantly increased in diabetic mice, and the inflammatory response in diabetic PFKFB3^wt/mut^ mice was greatly weakened compared to that in diabetic PFKFB3^wt/wt^ mice (Fig. 6C). CD68^+^ and CD206^+^ macrophages in diabetic PFKFB3^wt/mut^ mice showed similar changes compared with those in in diabetic IGFBP5^−/−^ mice (Fig. 6D). Consistently, the numbers of infiltrated M1 macrophages were decreased and M2 macrophages were increased in diabetic PFKFB3^wt/mut^ mice compared to diabetic PFKFB3^wt/wt^ mice (Fig. 6D, E). These results suggested that a mutation in PFKFB3 impaired macrophage recruitment to the glomeruli. Furthermore, to establish IGFBP5 overexpression in the kidneys of mice, tail vein injections of IGFBP5 overexpression lentivirus (lenti) or scramble (vehicle) lentivirus were performed (Fig. 6F, G). The development of mesangial expansion was significantly enhanced in lenti-treated diabetic PFKFB3^wt/wt^ mice but was markedly blunted in lenti-treated diabetic PFKFB3^wt/mut^ mice compared with vehicle-treated diabetic PFKFB3^wt/wt^ mice (Fig. 6H, I). These data indicate that a mutation in PFKFB3 markedly attenuates diabetic renal inflammation caused by IGFBP5.

### The PFKFB3 inhibitor 3PO suppresses the progression of DKD in db/db mice

**Fig. 7:**
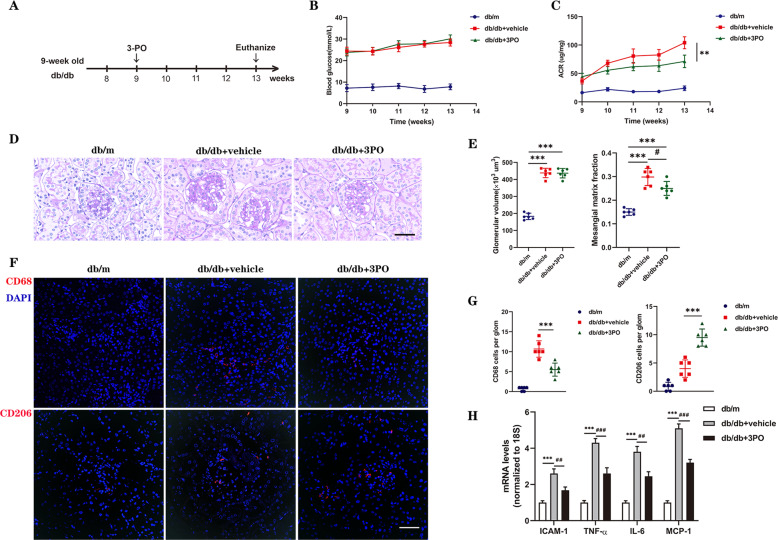
3PO suppresses the progression of glomerulopathy in db/db mice. **A** Schematics showing the experimental design. 3PO or DMSO was intraperitoneally injected into 9-week-old db/db and db/m mice. The mice were euthanized at 4 weeks postinjection for analysis (*n* = 6 mice per group). **B**, **C** Weekly blood glucose and ACR measurements of db/db and db/m mice. **D** Representative images of PAS-stained kidneys of db/db and db/m mice. Scale bar, 50 μm. **E** Quantification of the glomerular volume and mesangial matrix fraction per mouse; *n* = 6 mice. **F** Representative images of CD68 and CD206 immunofluorescence. Scale bar, 50 μm. **G** Quantification of CD68- and CD206-positive cells in db/db and db/m mice. **H** RT–PCR analysis of the mRNA levels of ICAM-1, TNF-α, IL-6, and MCP-1 in db/m and db/db mice. *n* = 6 mice. The data are shown as the mean ± SD. **P < 0.01, ***P < 0.001; ^#^*P* < 0.05, ^##^*P* < 0.01, ^###^*P* < 0.001.

Next, we investigated whether inhibiting glycolysis leads to a decrease in DKD development, and 3PO, a specific small-molecule inhibitor of PFKFB3, was administered to db/db mice intraperitoneally (Fig. 7A–C). No apparent side effects, such as decreases in body weight or systolic blood pressure (Fig. S1 and Table S3), were observed in 3PO-treated mice. In addition, the ACR of 3PO-treated db/db mice was significantly lower than that of DMSO-treated db/db mice (Fig. 7C), indicating that glomerular damage was blunted by 3PO treatment. Histopathological and morphometric analyses were performed. DMSO-treated db/db mice exhibited worsened mesangial matrix expansion, and this pathological feature was improved in 3PO-treated db/db mice (Fig. 7D, E). These data indicate that inhibiting PFKFB3 can improve renal pathology and glomerular injury in diabetic mice. CD68^+^ and CD206^+^ cells were counted. 3PO-treated db/db mice had significantly decreased CD68^+^ cell counts and increased CD206^+^ cell counts compared with those in vehicle-treated db/db mice (Fig. 7F, G). Consistent with the results from PFKFB3^wt/mut^ mouse kidney tissues, 3PO-mediated inhibition of PFKFB3 reduced the HG-induced increase in TNF-α, IL-6, ICAM-1, and MCP-1 mRNA levels (Fig. 7H). Thus, we revealed the key role of the glycolysis regulator PFKFB3 in the development of DKD in rodent models and found that inhibiting PFKFB3 inhibited the synthesis of inflammatory cytokines, chemokines, and adhesion molecules.

## Discussion

Metabolic pathways are not only vital in maintaining the energy balance but also critical in determining signaling pathways and cellular phenotype [43–45]. This study confirmed that IGFBP5 was related to renal inflammation in vitro and in vivo. A marked increase in glycolytic activity caused by IGFBP5 in ECs was mainly mediated by the glycolytic activator PFKFB3. Inhibiting PFKFB3 not only reversed the increase in glycolytic activity but also weakened the inflammatory response in ECs. Overall, these results indicated that the proinflammatory effect of IGFBP5 in the kidney was mediated by metabolic reprogramming in ECs.

Recent studies have indicated that metabolic reprogramming plays a pivotal role in the development of DKD [46–48]. However, most of these studies focused on podocytes and renal tubular epithelial cells in DKD, and few focused on metabolic changes in ECs. In this study, IGFBP5 induced profound glycolytic activation in ECs and promoted increases in glycolytic flux and glycolysis products, ultimately accelerating renal inflammation. Lactate, a product of glycolysis, has been regarded as a waste product. Recently, it has been reported that lactate acts as an inflammatory signal to promote chronic inflammation by driving T cell dysfunction and increasing the production of inflammatory cytokines [49–51]. Moreover, glycolysis can enhance NF-κB-driven vascular inflammation via lactate signaling [52, 53]. A previous study showed that lactate can modulate the phenotype of ECs [54]. However, the downstream effects of metabolic reprogramming and the key molecules in EC injury are not well understood. Thus, further research will focus on the mechanisms by which lactic acid accumulation in the kidney activates immune or inflammatory pathways and search for effective drugs to inhibit the pathological effects of lactic acid.

Intensive research has shown that macrophage infiltration is a prominent feature during the progression of chronic kidney disease. The single-cell RNA-sequencing analysis was performed on diabetic mice and revealed predominantly increased macrophage infiltration in glomeruli [55]. Macrophage infiltration is mediated by several chemokines and adhesion molecules, of which MCP-1 and ICAM-1 play major roles [56, 57]. Diabetes models have shown an influx of macrophages in response to increases in MCP-1 and ICAM-1 [58], and inhibiting this influx can reduce proteinuria [59]. Infiltrated macrophages release TNF-α, ROS, and proteases, all of which aggravate endothelial damage and promote the development of DKD [60, 61]. Macrophages are classified as classically activated M1 and alternatively activated M2 states [62]. Macrophages isolated from diabetic kidneys showed increased expression of M1 markers [63]. Our results showed that IGFBP5 promoted M1 macrophages and hindered M2 macrophage migration to the endothelium. After knocking down or deleting IGFBP5, the migration of M1 macrophages was obstructed, while that of M2 macrophages was significantly enhanced. Under chronic inflammatory conditions, such as DKD, both M1 and M2 macrophages exist [64], which was also confirmed by our data.

EGR1 is an early response transcription factor that regulates the expression of multiple downstream genes. Studies have shown that high expression of EGR1 resulting from glucose is involved in the pathological changes in diabetic retinopathy [65]. We demonstrated that IGFBP5 promoted the expression of EGR1, which bound to the PFKFB3 promoter, inducing the expression of PFKFB3 and enhancing glycolysis. A large number of studies have shown that endothelial PFKFB3-driven glycolysis regulates the inflammatory response [66, 67]. In the tumor microvasculature, PFKFB3 haploid defects reduce the expression of EC adhesion molecules by reducing NF-κB signal transduction [52]. In addition, increased arterial vascular endothelial glycolysis and enhanced inflammatory signaling can also be observed in pulmonary hypertension [29] and atherosclerosis [68]. Endothelial activation has been reported to be a hallmark of diabetes mellitus [10]. Our data showed that PFKFB3 was elevated in DKD and promoted glycolysis and the inflammatory response in ECs. Inhibiting PFKFB3 not only reversed the increase in glycolytic activity but also dampened endothelial inflammation and monocyte migration.

Inhibiting PFKFB3 damaged the inflammatory potential of ECs associated with IGFBP5. Our results showed that regulating metabolic states could be used to treat or even prevent DKD. Future studies using tissue-specific IGFBP5 knockout or overexpression mouse models are required to illustrate the role of IGFBP5 in DKD. IGFBP5 is a multifunctional protein that has various IGF-dependent and IGF-independent effects on different tissues and cells [69]. Previous study [70] and our work demonstrated elevated expression of IGFBP5 in ECs under HG conditions in vitro. It should be noted that to date, no specific IGFBP5 cell surface receptor has been identified. However, functional interactions with some cell surface proteins have been reported. For example, IGFBP5 directly interacts with α_2_β_1_ integrin on the surface of breast cancer cells to promote adhesion and inhibit migration [20]. Further studies are needed to elucidate the detailed mechanism by which IGFBP5 is upregulated in ECs in DKD and confirm the clinical implications of IGFBP5 in DKD patients.

In summary, this study explored the mechanism by which IGFBP5 regulated renal inflammation and showed that IGFBP5 enhanced renal inflammation. IGFBP5 activated endothelial inflammation, mainly through the activation of PFKFB3, thereby facilitating increased proinflammatory molecule production and macrophage migration. Silencing IGFBP5 blunted the inflammatory potential of ECs in DKD. Our study confirms a new proinflammatory role of IGFBP5 in the pathogenesis of DKD through PFKFB3, suggesting that IGFBP5 may be a promising target for the treatment of DKD to inhibit early inflammatory responses and delay DKD progression.

## Supplementary information

Supplementary Material

western blots

aj-checklist

## Data Availability

The data that support the findings of this study are available on request from the corresponding author.
