# Supplementary material for: IGFBP5 promotes diabetic kidney disease progression by enhancing PFKFB3-mediated endothelial glycolysis
Source: Cell Death Dis. 2022 Apr 13;13(4):340. doi: 10.1038/s41419-022-04803-y (PMC9007962; doi:10.1038/s41419-022-04803-y)
Supplement: Supplementary file 1 — Supplementary Material [file 41419_2022_4803_MOESM1_ESM.docx]

Supplementary material for

**IGFBP5 promotes diabetic kidney disease progression by enhancing PFKFB3-mediated endothelial glycolysis**

Chengcheng Song^1,2^, Shuqiang Wang^2,3^, Zhangning Fu^1,2^, Kun Chi^1,2^, Xiaodong Geng^2,4^, Chao Liu^2^, Guangyan Cai^2^, Xiangmei Chen^2^, Di Wu^2*^, and Quan Hong^2*^

^1^ Medical School of Chinese PLA, Beijing 100853, China

^2^ Department of Nephrology, First Medical Center of Chinese PLA General Hospital, Nephrology Institute of the Chinese People's Liberation Army, State Key Laboratory of Kidney Diseases, National Clinical Research Center for Kidney Diseases, Beijing Key Laboratory of Kidney Disease Research, Beijing 100853, China

^3^ Department of Nephrology, Peking University Shenzhen Hospital, Shenzhen, 518000, China

^4^ Beidaihe Rehabilitation and Recuperation Center, Chinese People’s Liberation Army Joint Logistics Support Force, Qinhuangdao 066100, China

**Correspondence:**

*Quan Hong, PhD, Department of Nephrology, State Key Laboratory of Kidney Diseases,

Chinese PLA General Hospital, +86-01066937087, hongquan@301.hospital.com.cn

*Di Wu, PhD, Department of Nephrology, State Key Laboratory of Kidney Diseases, Chinese PLA General Hospital, +86-01063503586, 13213875@qq.com

***Quan Hong and Di Wu shared the corresponding authorship in this work.**

**Author contributions**

QH and DW supervised the project, designed, edited, and led out the experiments of this study. GYC and XMC revised the manuscript. CCS and SQW drafted the manuscript. CCS, ZNF and KC organized the data and prepared all the figures and tables. CCS, XDG and CL conducted the experiments and data analysis.

**Supplemental methods**

**Lentivirus-mediated gene overexpression**

Stable IGFBP5 overexpression HUVEC lines (IGFBP5-OE) were established using lentivirus infection and IGFBP5 overexpression sequence, which was constructed by Hunan Fenghui Biotechnology Co., Ltd. A recombinant lentivirus and negative control lentivirus (vehicle) were prepared and titered to 10^8^ transfection units/ml. To obtain stably transfected IGFBP5-OE cells, HUVECs were seeded in six-well dishes at a density of 1 x 10^5^ cells per well. The cells were then infected with the same virus titer on the next day. At 72 h post-viral infection, the culture medium was replaced with a selection medium containing 1 μg/ml puromycin (Beyotime, Shanghai China). The puromycin-resistant cells were amplified in a medium containing 1 μg/ml puromycin for 5 days. Finally, positive clones were verified by RT-PCR.

**Supplemental figures**


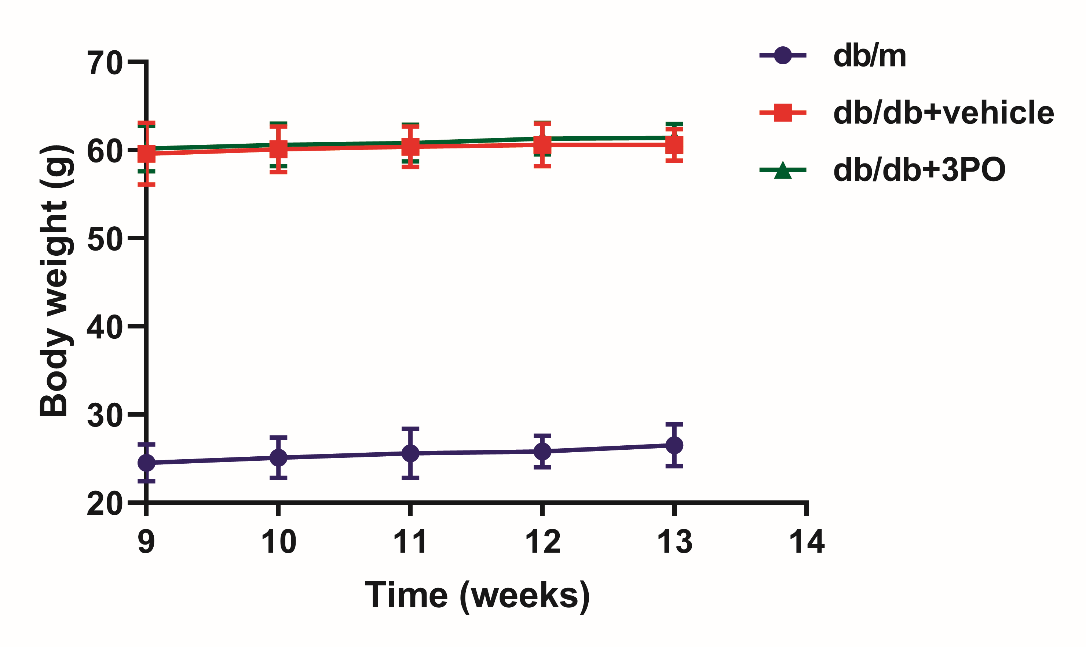


Fig. S1: Body weight measurements in db/db and db/m mice.

**Supplemental tables**

**Table S1. Sequences of siRNAs used.**

|  | Target gene | Species |  | Sequence (5′-3′) |
| --- | --- | --- | --- | --- |
| siRNA | IGFBP5 | Human | Forward | GCUGUGUACCUGCCCAAUUTT |
|  |  |  | Reverse | AAUUGGGCAGGUACACAGCTT |
| siRNA | PFKFB3 | Human | Forward | GAGAUGCCCUACCUGAAAUTT |
|  |  |  | Reverse | AUUUCAGGUAGGGCAUCUCTT |
| siRNA | CTRL(Non-targeting) | Human | Forward | UUCUCCGAACGUGUCACGUTT |
|  |  |  | Reverse | ACGUGACACGUUCGGAGAATT |

**Table S2. Primer sequences used for qRT-PCR analyses.**

| Primer (Human) |  | Sequence (5′-3′) |
| --- | --- | --- |
| IGFBP5 | Forward | CCCAATTGTGACCGCAAAGG |
|  | Reverse | GGCAGCTTCATCCCGTACTT |
| PFKFB3 | Forward | CAGCTGCCTGGACAAAACAT |
|  | Reverse | CGTCTGCCTCAGTGTTTCCT |
| EGR1 | Forward | CTGACCGCAGAGTCTTTTCCTG |
|  | Reverse | TGGGTGCCGCTGAGTAAATG |
| TNF-α | Forward | CACAGTGAAGTGCTGGCAAC |
|  | Reverse | GATCAAAGCTGTAGGCCCCA |
| ICAM-1 | Forward | AGCTTCGTGTCCTGTATGGC |
|  | Reverse | TTTTCTGGCCACGTCCAGTT |
| IL-6 | Forward | CCACCGGGAACGAAAGAGAA |
|  | Reverse | GAGAAGGCAACTGGACCGAA |
| MCP-1 | Forward | ACAAGCAAACCCAAACTCCG |
|  | Reverse | AACAGGGTGTCTGGGGAAAG |
| 18S | Forward | GTAACCCGTTGAACCCCATT |
|  | Reverse | CCATCCAATCGGTAGTAGCG |
| Primer (Mouse) |  | **Sequence (5′-3′)** |
| IGFBP5 | Forward | CCCAATTGTGACCGCAAAGG |
|  | Reverse | GGCAGCTTCATCCCGTACTT |
| PFKFB3 | Forward | GGGAGAGGTCAGAGAACATGAA |
|  | Reverse | GGAACCCACATCTCGGCTTT |
| TNF-α | Forward | AGGGTCTGGGCCATAGAACT |
|  | Reverse | CCACCACGCTCTTCTGTCTAC |
| ICAM-1 | Forward | GTGGCGGGAAAGTTCCTG |
|  | Reverse | CGTCTTGCAGGTCATCTTAGGAG |
| IL-6 | Forward | TCTTGGGACTGATGCTGGTGA |
|  | Reverse | GCAAGTGCATCATCGTTGTTCA |
| MCP-1 | Forward | TTAAAAACCTGGATCGGAACCAA |
|  | Reverse | GCATTAGCTTCAGATTTACGGGT |

**Table S3. Blood pressure measurements of db/db and db/m mice.**

| mmHg | db/m | db/db | db/db+3PO |
| --- | --- | --- | --- |
| SBP (mean ± SEM) | 102.5±6.2 | 108.2±12.3 | 110.2±10.3 |
| DBP (mean ± SEM) | 54.7**±**8.4 | 60.4**±**15.1 | 59.3**±**14.2 |

Blood pressure was measured by tail-cuff blood pressure monitor at 4 weeks post vehicle or 3PO injections.
