# Supplementary material for: IGFBP5 promotes diabetic kidney disease progression by enhancing PFKFB3-mediated endothelial glycolysis
Source: Cell Death Dis. 2022 Apr 13;13(4):340. doi: 10.1038/s41419-022-04803-y (PMC9007962; doi:10.1038/s41419-022-04803-y)
Supplement: Supplementary file 2 — western blots [file 41419_2022_4803_MOESM2_ESM.pptx]

## Slide 1
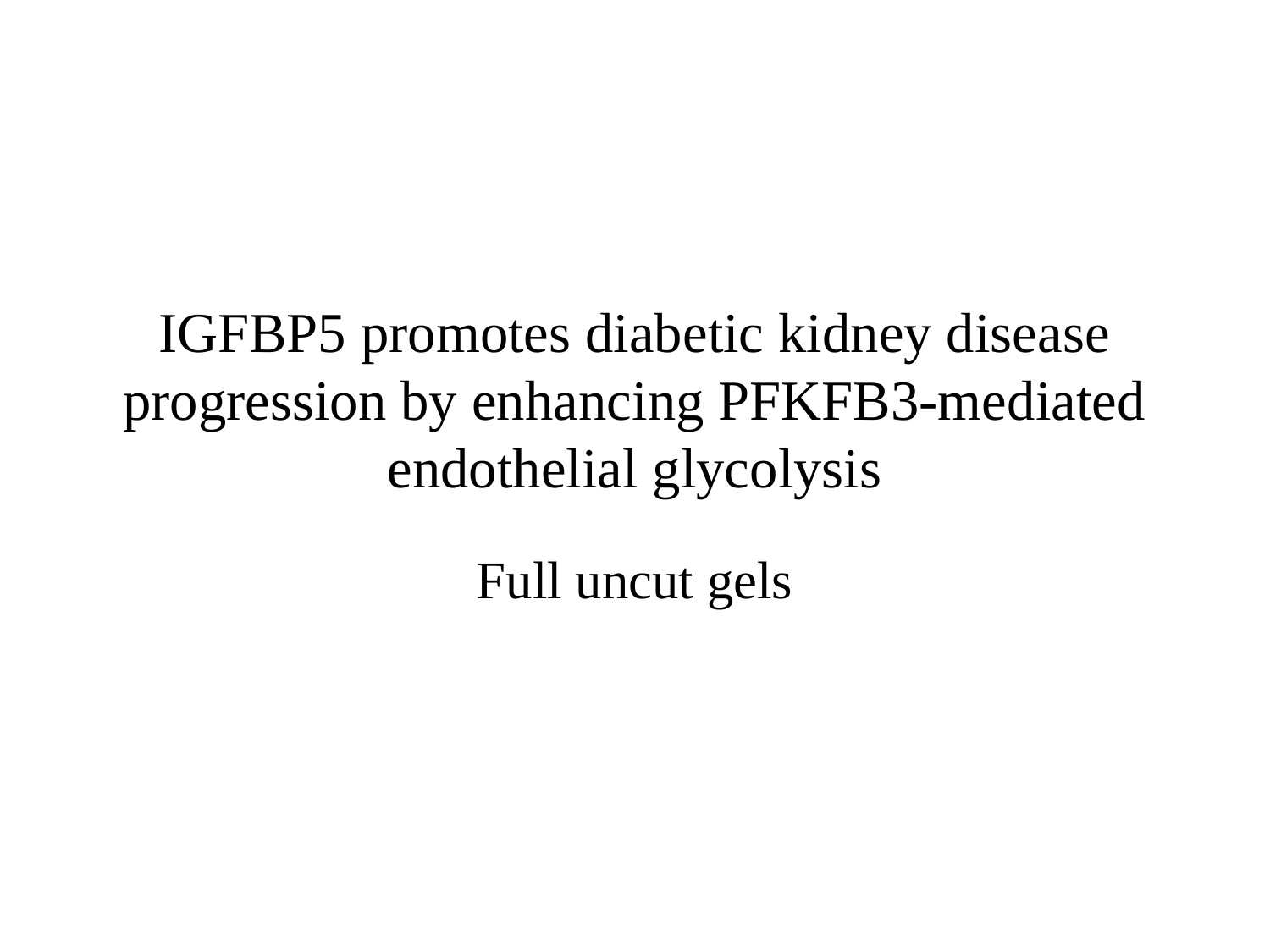

# IGFBP5 promotes diabetic kidney disease progression by enhancing PFKFB3-mediated endothelial glycolysis
Full uncut gels

## Slide 2
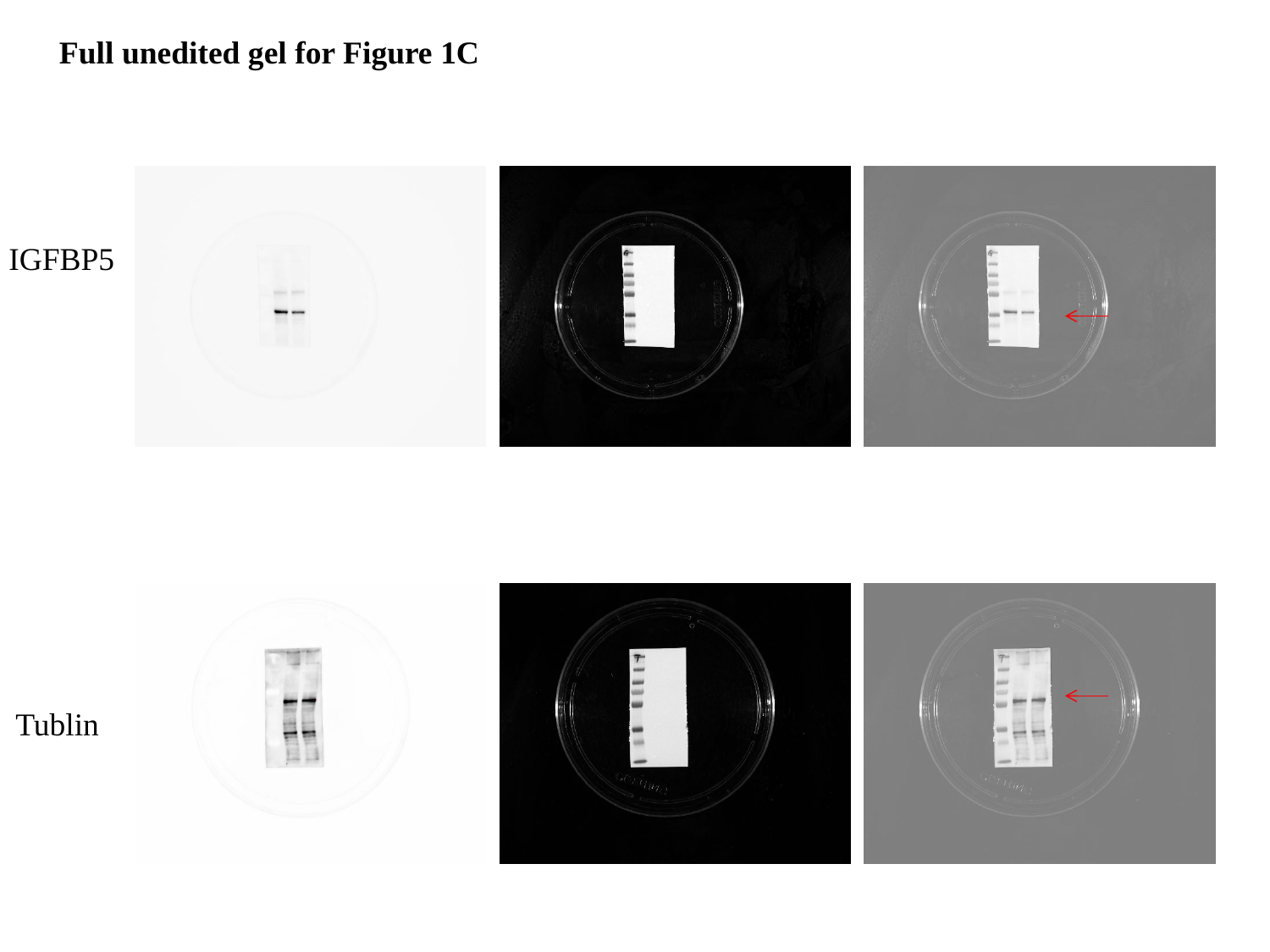

Full unedited gel for Figure 1C
IGFBP5
Tublin

## Slide 3
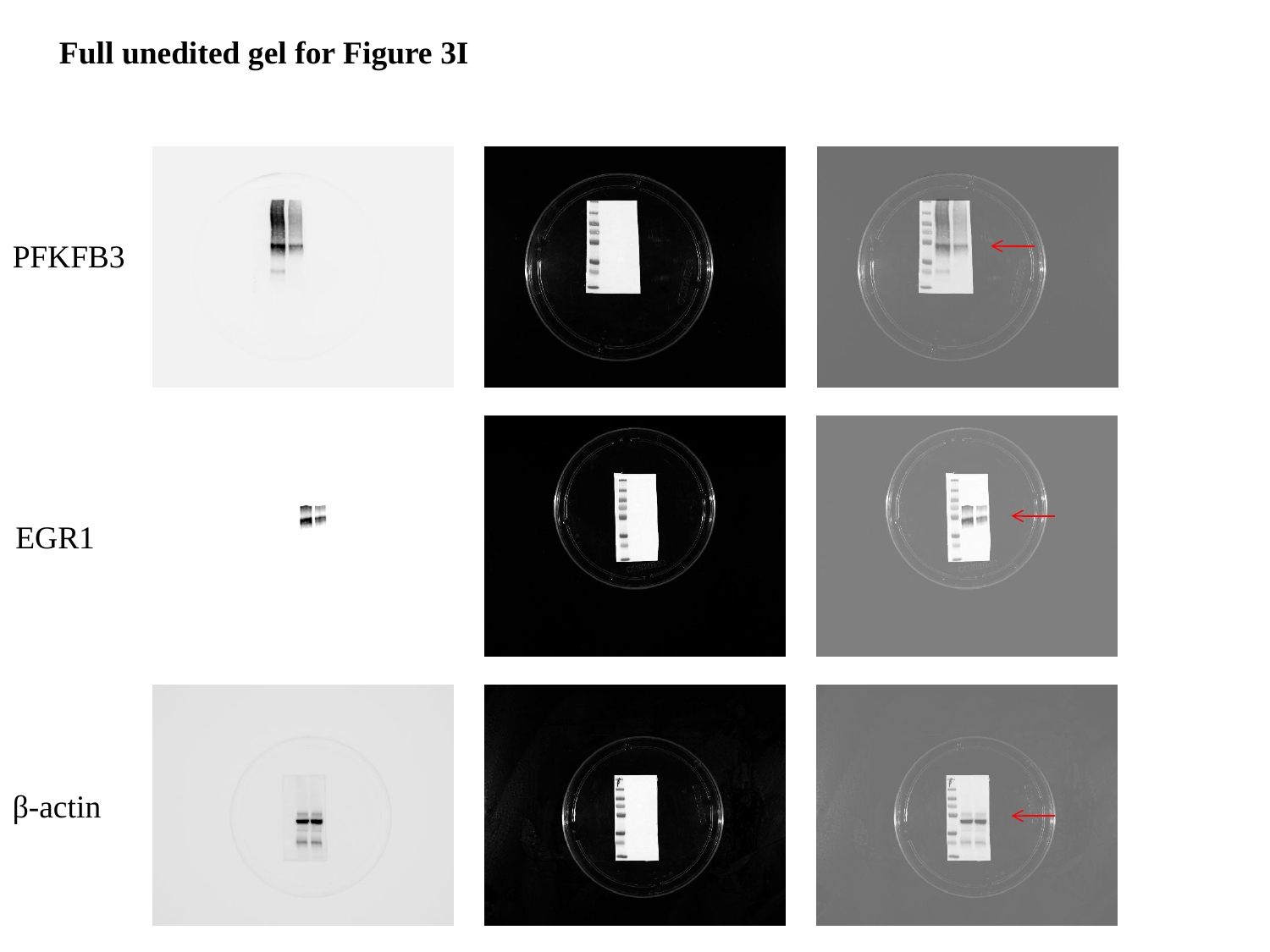

Full unedited gel for Figure 3I
PFKFB3
EGR1
β-actin

## Slide 4
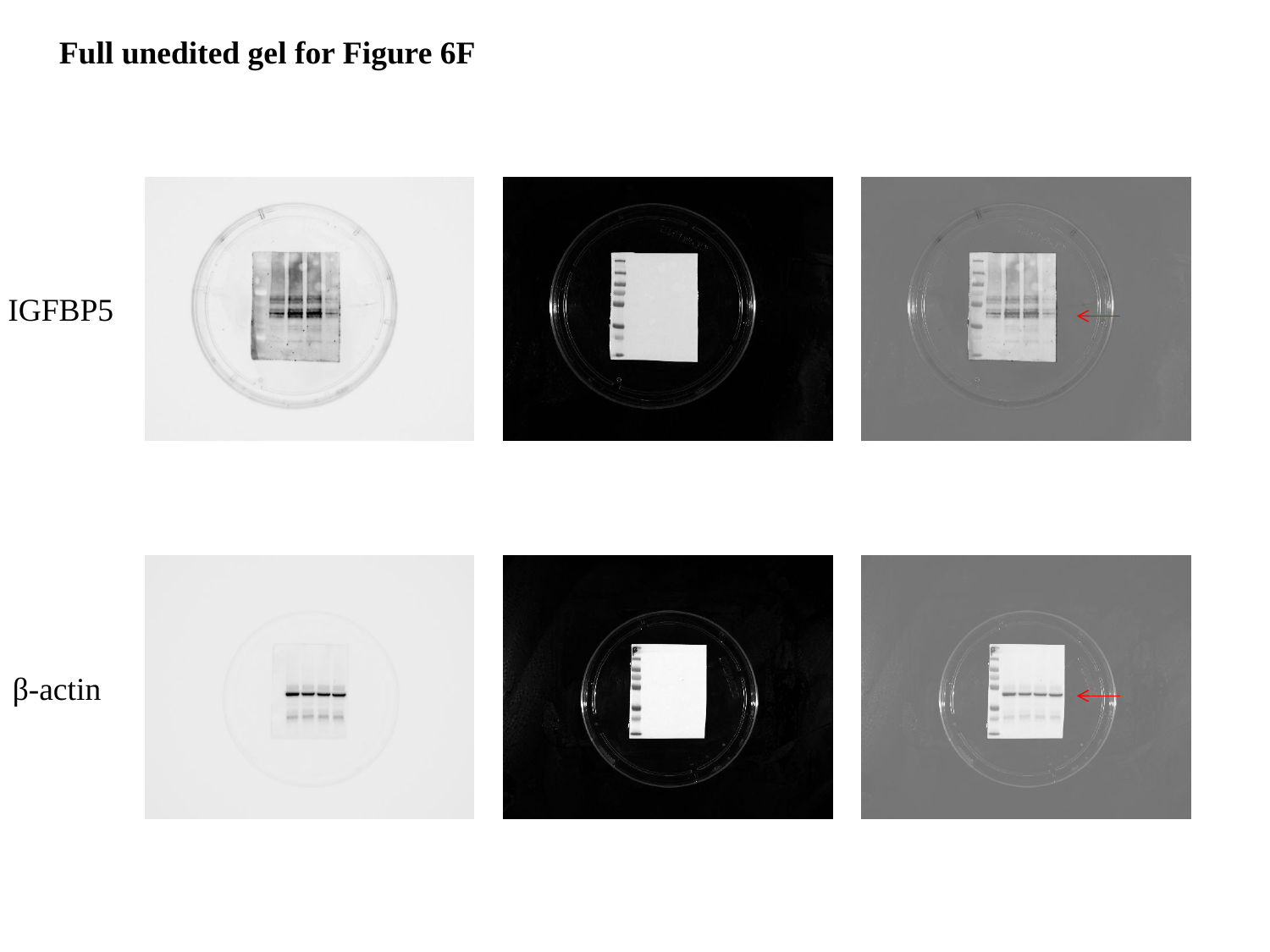

Full unedited gel for Figure 6F
IGFBP5
β-actin
